# Supplementary material for: Burden of soil-transmitted helminth infection in pregnant refugees and migrants on the Thailand-Myanmar border: Results from a retrospective cohort
Source: PLoS Negl Trop Dis. 2021 Mar 1;15(3):e0009219. doi: 10.1371/journal.pntd.0009219 (PMC7951971; doi:10.1371/journal.pntd.0009219)
Supplement: S1 Fig — (DOCX) [file pntd.0009219.s001.docx]

# S1 Fig. Proportion of anaemia at the first ANC contact in relation to the infection intensity and compared between STH monoinfections (pooled data).

|  |
| --- |
| Proportion of cases presenting with anaemia at the first ANC contact compared between STH negative cases, cases with a rare, low, mild and high infection intensity respectively.  Data shown as percentage (%). The error bars indicate the 95% confidence interval.  Abbreviations: ANC, antenatal care; STH, soil-transmitted helminth. |
