# Supplementary material for: Burden of soil-transmitted helminth infection in pregnant refugees and migrants on the Thailand-Myanmar border: Results from a retrospective cohort
Source: PLoS Negl Trop Dis. 2021 Mar 1;15(3):e0009219. doi: 10.1371/journal.pntd.0009219 (PMC7951971; doi:10.1371/journal.pntd.0009219)
Supplement: S1 Table — (DOCX) [file pntd.0009219.s002.docx]

# S1 Table. Comparison of included and excluded cases

Distribution of demographic and clinical characteristics between included and excluded (i.e. no stool exam available) women attending SMRU antenatal clinics.

|  | Included (n=12,742) | Excluded (n=2,304) |
| --- | --- | --- |
| Age (years), median (IQR) | 25 (20-31) | 26 (21-32)^$^ |
| Age Group, n (%) |  |  |
| - <20 | 2,228 (17.5) | 402 (17.4) |
| - 20-29 | 6,596 (51.8) | 1,079 (46.8) |
| - 30-39 | 3,374 (26.5) | 659 (28.6) |
| - ≥ 40 | 544 (4.3) | 162 (7.0) |
| Underweight, n (%) | 1,278 (10.0) † | 242 (10.5) †† |
| Short stature, n (%) | 1,294 (10.2) ‡ | 249 (10.8) ‡‡ |
| Site, n (%) |  |  |
| - Migrant site | 8,701/10,749 (80.9) | 2,048/10,749 (19.1) |
| - Refugee camp | 4,041/4,297 (94.0) | 256/4,297 (6.0) |
| No. of previous pregnancies, n (%) |  |  |
| - Primigravida, n (%) | 4,272 (33.5) | 717 (31.1) |
| - 2 | 2,981 (23.4) | 475 (20.6) |
| - 3 | 2,049 (16.1) | 315 (13.7) |
| - ≥ 4 | 3,440 (27.0) | 797 (34.6) |
| First ANC in trimester 1, n (%) | 5,052 (39.6) | 748 (32.5) |
| Literate, n (%) | 7,919 (62.1) | 1,323 (57.4)^$^ |
| Smoker, n (%) | 1,439 (11.3) | 367 (15.9)^$^ |
| Anaemia first HCT, n (%) | 668 (5.2) ¶ | 160 (7.1) ¶¶ |
| Data shown as proportions n (%) or median (IQR).  Of the 4,882 excluded women, 1,109 did not provide a stool test, for 2,578 women the pregnancy outcome was unknown and for 1,195 no stool test was available and pregnancy outcome was unknown.  $ 2 missing cases.  † 6 missing cases, †† 52 missing cases.  ‡ 4 missing case, ‡‡ 37 missing cases.  ¶ 6 missing cases, ¶¶ 43 missing cases. | | |
