# Supplementary material for: Burden of soil-transmitted helminth infection in pregnant refugees and migrants on the Thailand-Myanmar border: Results from a retrospective cohort
Source: PLoS Negl Trop Dis. 2021 Mar 1;15(3):e0009219. doi: 10.1371/journal.pntd.0009219 (PMC7951971; doi:10.1371/journal.pntd.0009219)
Supplement: S2 Table — (DOCX) [file pntd.0009219.s003.docx]

# S2 Table. Time trend of infected pregnant women attending the migrant and refugee ANC clinics

|  | Migrants (n=8,701) | | | | | Refugees (n=4,041) | | | | |
| --- | --- | --- | --- | --- | --- | --- | --- | --- | --- | --- |
| **Total,**  n (%) | STH neg  7,245 (83.3) | HW MI  592 (6.8) | AL MI  470 (5.4) | TT MI  210 (2.4) | Co-infection  184 (2.1) | STH neg  2,795 (69.2) | HW MI  69 (1.7) | AL MI  943 (23.3) | TT MI  102 (2.5) | Co-infection  132 (3.3) |
| 2013* | 654 (76.8) | 90 (10.6) | 61 (7.2) | 25 (2.9) | 22 (2.6) | 481 (70.2) | 16 (2.3) | 140 (20.4) | 22 (3.2) | 26 (3.8) |
| 2014 | 1,566 (81.0) | 154 (8.0) | 104 (5.4) | 57 (2.9) | 53 (2.7) | 846 (64.5) | 30 (2.3) | 340 (25.9) | 37 (2.8) | 58 (4.4) |
| 2015 | 1,597 (82.6) | 124 (6.4) | 129 (6.7) | 50 (2.6) | 34 (1.8) | 863 (71.3) | 15 (1.2) | 280 (23.1) | 25 (2.1) | 27 (2.2) |
| 2016 | 1,762 (86.2) | 114 (5.6) | 98 (4.8) | 39 (1.9) | 31 (1.5) | 605 (72.5) | 8 (1.0) | 183 (21.9) | 18 (2.2) | 21 (2.5) |
| 2017^$^ | 1,666 (86.0) | 110 (5.7) | 78 (4.0) | 39 (2.0) | 44 (2.3) | NA | NA | NA | NA | NA |
| Intensity of infection, n (%) | |  |  |  |  |  |  |  |  |  |
| - Rare | | 399 (67.4) | 132 (28.1) | 150 (71.4) | NA |  | 35 (50.7) | 242 (25.7) | 69 (67.6) | NA |
| - Low | | 150 (25.3) | 170 (36.2) | 47 (22.4) | NA |  | 19 (27.5) | 214 (22.7) | 27 (26.5) | NA |
| - Medium | | 34 (5.7) | 107 (22.8) | 11 (5.2) | NA |  | 8 (11.6) | 254 (27.0) | 6 (5.9) | NA |
| - High | | 9 (1.5) | 61 (13.0) | 2 (1.0) | NA |  | 7 (10.1) | 233 (24.7) | 0 | NA |
| Data shown as proportions n (%).  * Data available for 6 months (July-December) as routine screening for intestinal parasitic infections commenced mid 2013.  $ As operations ceased in the refugee camp at the end of 2016, no data is available for the refugee population for 2017. Abbreviations: AL, *Ascaris lumbricoides*; HW, *hookworm*; STH, soil transmitted helminth; TT, *Trichuris trichiura* | | | | | | | | | | |
