# Supplementary material for: Burden of soil-transmitted helminth infection in pregnant refugees and migrants on the Thailand-Myanmar border: Results from a retrospective cohort
Source: PLoS Negl Trop Dis. 2021 Mar 1;15(3):e0009219. doi: 10.1371/journal.pntd.0009219 (PMC7951971; doi:10.1371/journal.pntd.0009219)
Supplement: S3 Table — (DOCX) [file pntd.0009219.s004.docx]

# S3A Table. Associations between anaemia at first ANC contact and a STH infection in the migrant population

|  | No Anaemia (n=8,114) | Anaemia (n=400) | Unadjusted OR (95% CI) | p-value | Adjusted OR (95% CI)^$^ | p-value^$^ | PAF (%) | |
| --- | --- | --- | --- | --- | --- | --- | --- | --- |
| **STH infections** |  |  |  |  |  |  |  | |
| STH neg | 6,913 (95.5) | 329 (4.5) | Referent |  | Referent |  |  | |
| STH category |  |  |  |  |  |  | 2.7 (-1.6-7.0) | |
| HW MI | 554 (93.6) | 38 (6.4) | 1.44 (1.00-2.01) | 0.039 | 1.42 (0.98-2.00) | 0.051 |  | |
| AL MI | 448 (95.3) | 22 (4.7) | 1.03 (0.64-1.57) | 0.889 | 0.93 (0.58-1.42) | 0.743 |  | |
| TT MI | 199 (94.8) | 11 (5.2) | 1.16 (0.59-2.05) | 0.634 | 1.23 (0.62-2.19) | 0.523 |  | |
| Age Group (years) |  |  |  |  |  |  |  | |
| - <20 | 1,418 (96.3) | 54 (3.7) | Referent |  | Referent |  |  | |
| - 20-29 | 4,281 (96.2) | 171 (3.8) | 1.05 (0.77-1.44) | 0.764 | 0.90 (0.64-1.27) | 0.533 | -4.7 (-19.8-10.4) | |
| - 30-39 | 2,073 (93.7) | 139 (6.3) | 1.76 (1.28-2.45) | <0.001 | 1.26 (0.85-1.87) | 0.253 | 6.7 (-4.1-17.4) | |
| - ≥ 40 | 342 (90.5) | 36 (9.5) | 2.76 (1.77-4.27) | <0.001 | 1.88 (1.13-3.09) | 0.014 | 3.9 (0.6-7.2) | |
| Trimester 1^st^ ANC contact |  |  |  |  |  |  |  | |
| - 1^st^ Trimester | 2,949 (98.2) | 53 (1.8) | Referent | Referent |  |  |  | |
| - 2^nd^ Trimester | 4,011 (94.1) | 253 (5.9) | 3.51 (2.62-4.78) | <0.001 | 3.50 (2.60-4.78) | <0.001 | 44.1 (35.9-52.3) | |
| - 3^rd^ Trimester | 1,154 (92.5) | 94 (7.5) | 4.53 (3.23-6.43) | <0.001 | 4.58 (3.24-6.55) | <0.001 | 17.9 (13.3-22.5) | |
| Smoker | 795 (9.8) | 79 (19.8) | 2.27 (1.74-2.91) | <0.001 | 1.73 (1.31-2.27) | <0.001 | 7.8 (3.4-12.2) | |
| Multigravida | 5,264 (64.9) | 299 (74.8) | 1.60 (1.28-2.03) | <0.001 | 1.26 (0.95-1.68) | 0.106 | 14.3 (-2.4-31.0) | |
| BMI Underweight^‡^ | 877 (10.8) | 36 (9.0) | 0.82 (0.57-1.14) | 0.254 | 1.20 (0.82-1.70) | 0.325 | 1.4 (-1.6-4.4) | |
| Malaria 1^st^ ANC | 54 (0.7) | 9 (2.3) | 3.44 (1.57-6.66) | <0.001 | 3.21 (1.45-6.37) | 0.002 | 1.4 (0.0-2.8) | |
| Intensity of infection |  |  |  |  |  |  |  | |
| - STH neg | 6,913 (95.5) | 329 (4.5) | Referent |  |  |  |  | |
| - Rare | 647 (95.0) | 34 (5.0) | 1.10 (0.76-1.56) | 0.592 |  |  |  | |
| - Low | 346 (94.3) | 21 (5.7) | 1.28 (0.79-1.96) | 0.294 |  |  |  | |
| - Medium | 143 (94.1) | 9 (5.9) | 1.32 (0.62-2.47) | 0.422 |  |  |  | |
| - High | 65 (90.3) | 7 (9.7) | 2.26 (0.94-4.64) | 0.042 |  |  |  | |
| Data displayed as n (%), odds ratios and 95% confidence interval for unadjusted and adjusted models, based on logistic regression.  Cases with a known haematocrit and either a negative stool sample or a HW-, AL- or TT monoinfection were included in this analysis (3 cases without a HCT sample and 184 cases with a multiple infection were removed)  Exposure to STHs were estimated for monoinfections of HW, AL and TT, and compared to STH negative pregnant women.  ‡ defined as BMI <18.5 kg/m^2^ according to Asian BMI groups.  § defined as HCT<30%.  $ adjusted for (i) STH categories, (ii) migration status, (iii) smoking, (iv) age, (v) gravidity (primi- vs multigravida), (vi), trimester first ANC contact, (vii), underweight, (viii) malaria 1^st^ ANC contact.  Abbreviations: ANC, antenatal care; AL, *Ascaris lumbricoides*; BMI, body-mass index; CI, confidence interval; HW, hookworm; MI, monoinfection; neg, negative; OR, odds ratio; PAF, population attributable fraction; STH, soil-transmitted helminth; TT, *Trichuris trichiura* | | | | | | | |  |

# S3B Table. Associations between anaemia at first ANC contact and a STH infection in the refugee population

|  | No Anaemia (n=3,656) | Anaemia (n=250) | Unadjusted OR (95% CI) | p-value | Adjusted OR (95% CI)^$^ | p-value^$^ | PAF (%) | |
| --- | --- | --- | --- | --- | --- | --- | --- | --- |
| **STH infections** |  |  |  |  |  |  |  | |
| STH neg | 2,642 (94.6) | 151 (5.4) | Referent |  | Referent |  |  | |
| STH category |  |  |  |  |  |  | 14.4 (6.3-22.5) | |
| HW MI | 57 (83.8) | 11 (16.8) | 3.38 (1.65-6.33) | <0.001 | 3.06 (1.45-5.94) | 0.002 |  | |
| AL MI | 860 (91.2) | 83 (8.8) | 1.69 (1.27-2.23) | <0.001 | 1.70 (1.26-2.27) | <0.001 |  | |
| TT MI | 97 (95.1) | 5 (4.9) | 0.90 (0.31-2.03) | 0.825 | 0.80 (0.27-1.85) | 0.635 |  | |
| Age Group (years) |  |  |  |  |  |  |  | |
| - <20 | 650 (94.3) | 39 (5.7) | Referent |  | Referent |  |  | |
| - 20-29 | 1,874 (94.6) | 107 (5.4) | 0.95 (0.66-1.40) | 0.797 | 0.84 (0.55-1.31) | 0.442 | -6.7 (-24.5-11.1) | |
| - 30-39 | 996 (92.2) | 84 (7.7) | 1.41 (0.96-2.10) | 0.089 | 1.09 (0.66-1.80) | 0.738 | 2.2 (-10.8-15.2) | |
| - ≥ 40 | 136 (87.2) | 20 (12.2) | 2.45 (1.36-4.29) | 0.002 | 1.88 (0.94-3.69) | 0.069 | 3.2 (-0.4-6.8) | |
| Trimester 1^st^ ANC contact |  |  |  |  |  |  |  | |
| - 1^st^ Trimester | 1,894 (98.4) | 31 (1.6) | Referent | Referent |  |  |  | |
| - 2^nd^ Trimester | 1,341 (90.1) | 147 (9.9) | 6.70 (4.58-10.1) | <0.001 | 6.71 (4.47-10.08) | <0.001 | 49.1 (41.0-57.1) | |
| - 3^rd^ Trimester | 421 (85.4) | 72 (14.6) | 10.4 (6.84-16.3) | <0.001 | 10.52 (6.67-16.60) | <0.001 | 25.6 (19.7-31.5) | |
| Smoker | 449 (12.3) | 70 (28.0) | 2.78 (2.06-3.71) | <0.001 | 1.88 (1.34-2.60) | <0.001 | 11.2 (4.7-17.6) | |
| Multigravida | 2,499 (68.4) | 184 (73.6) | 1.29 (0.97-1.74) | 0.084 | 1.11 (0.77-1.62) | 0.576 | 7.2 (-13.5-28.0) | |
| BMI Underweight^‡†^ | 319 (8.7) | 12 (4.8)† | 0.53 (0.28-0.92) | 0.035 | 1.11 (0.57-1.99) | 0.744 | 0.5 (-2.2-3.2) | |
| Malaria 1^st^ ANC | 7 (0.2) | 6 (2.4) | 12.80 (4.10-38.90) | <0.001 | 6.85 (2.13-21.4) | <0.001 | 1.8 (0.0-3.5) | |
| Intensity of infection |  |  |  |  |  |  |  | |
| - STH neg | 2,642 (94.6) | 151 (5.4) | Referent |  |  |  |  | |
| - Rare | 318 (91.9) | 28 (8.1) | 1.54 (0.99-2.31) | 0.044 |  |  |  | |
| - Low | 234 (90.3) | 25 (9.7) | 1.87 (1.17-2.86) | 0.006 |  |  |  | |
| - Medium | 247 (92.2) | 21 (7.8) | 1.49 (0.90-2.34) | 0.101 |  |  |  | |
| - High | 215 (89.6) | 25 (10.4) | 2.03 (1.28-3.12) | 0.002 |  |  |  | |
| Data displayed as n (%), odds ratios and 95% confidence interval for unadjusted and adjusted models, based on logistic regression.  Cases with a known haematocrit and either a negative stool sample or a HW-, AL- or TT monoinfection were included in this analysis (3 cases without a HCT sample and 132 cases with a multiple infection were removed)  Exposure to STHs were estimated for monoinfections of HW, AL and TT, and compared to STH negative pregnant women.  ‡ defined as BMI <18.5 kg/m^2^ according to Asian BMI groups.  § defined as HCT<30%.  † 1 case with missing information.  $ adjusted for (i) STH categories, (ii) migration status, (iii) smoking, (iv) age, (v) gravidity (primi- vs multigravida), (vi), trimester first ANC contact, (vii), underweight, (viii) malaria 1^st^ ANC contact.  Abbreviations: ANC, antenatal care; AL, *Ascaris lumbricoides*; BMI, body-mass index; CI, confidence interval; HW, hookworm; MI, monoinfection; neg, negative; OR, odds ratio; PAF, population attributable fraction; STH, soil-transmitted helminth; TT, *Trichuris trichiura* | | | | | | | |  |
