# Supplementary material for: Burden of soil-transmitted helminth infection in pregnant refugees and migrants on the Thailand-Myanmar border: Results from a retrospective cohort
Source: PLoS Negl Trop Dis. 2021 Mar 1;15(3):e0009219. doi: 10.1371/journal.pntd.0009219 (PMC7951971; doi:10.1371/journal.pntd.0009219)
Supplement: S4 Table — (DOCX) [file pntd.0009219.s005.docx]

# S4 Table. Pregnancy outcome compared between STH infections in migrants and refugees.

|  | Migrants | | | | Refugees | | | |
| --- | --- | --- | --- | --- | --- | --- | --- | --- |
|  | STH neg | HW MI | AL MI | TT MI | STH neg | HW MI | AL MI | TT MI |
| Miscarriage*, % (n/N) | 12.9 (265/2,048) | 9.5 (17/179) | 12.6 (12/95) | 5.2 (3/58) | 17.0 (196/1,151) | 33.3 (8/24) | 11.0 (47/428) | 20.0 (8/40) |
| Stillbirth^#^, % (n/N) | 1.0 (50/5,247) | 0.9 (4/461) | 0.9 (3/330) | 0.6 (1/163) | 1.0 (22/2,139) | 0 (0) | 0.9 (7/781) | 1.2 (1/80) |
| Preterm birth^‡^, % (n/N) | 5.6 (287/5,090) | 8.2 (37/450) | 5.1 (16/316) | 5.0 (8/159) | 7.1 (146/2,065) | 6.2 (3/48) | 6.1 (47/768) | 9.0 (7/78) |
| Small for gestational age^$†^, % (n/N) | 20.6 (918/4,462) | 21.7 (84/387) | 16.2 (45/278) | 17.6 (26/148) | 16.3 (321/1,964) | 23.3 (10/43) | 15.4 (111/722) | 17.3 (13/75) |
| Data displayed as proportion (%) of cases (n) in relation to the total number (N).  * defined as pregnancy outcome before completion of 28 weeks of gestation; only cases who presented in first trimester included.  # defined as pregnancy outcome after completion of 28 weeks of gestation and neonate without any signs of life.  ‡ defined as pregnancy outcome after 28 weeks of gestation and before completion of 37 weeks of gestation with a liveborn neonate. Cases who had first ANC contact after 37 weeks of gestation excluded.  $ cases included if birthweight was measured within the first 72 hours of life; neonates with congenital abnormalities were excluded.  † defined as birthweight below the 10^th^ centile, following gestation adjusted birthweight centiles (Intergrowth 21^st^).  Abbreviations: AL, *Ascaris Lumbricoides*; HW, hookworm; MI, monoinfection; STH, soil-transmitted helminths; TT, *Trichuris trichiura*. | | | | | | | | |

|  |
| --- |
