# Supplementary material for: Burden of soil-transmitted helminth infection in pregnant refugees and migrants on the Thailand-Myanmar border: Results from a retrospective cohort
Source: PLoS Negl Trop Dis. 2021 Mar 1;15(3):e0009219. doi: 10.1371/journal.pntd.0009219 (PMC7951971; doi:10.1371/journal.pntd.0009219)
Supplement: S1 Text — (DOCX) [file pntd.0009219.s006.docx]

# S1 Text. Statistical methods for the estimation of the association between a soil-transmitted helminth infection and pregnancy outcome

To estimate the association between exposure to STH monoinfections and birth outcomes, Cox proportional hazards (outcomes - miscarriage and stillbirth) and logistic (outcomes - PTB and SGA) regression analyses were performed on a dataset that pooled outcomes in the migrant an refugee population. To select potential confounders for model adjustment, content knowledge and the package *DAGitty* (Version 3.0) were used to identify causal biological pathways between explanatory variables and birth outcomes [1].

Associations between STH and pregnancy outcomes are presented as adjusted hazards or odds ratio in a forest plot (Figure 3).

## Association of STH infections and miscarriage

To investigate the association of STH infections and miscarriage (i.e. pregnancy loss before completion of 28 weeks of gestation), a Cox regression model was chosen to account for variable follow-up times. The data to investigate pregnancy loss is left truncated as pregnancy loss may occur before a woman presents to antenatal care. Moreover, the risk of fetal loss changes with the progression of the pregnancy [2]. Hence the Cox proportional hazards model that was fitted for miscarriage accounted for left truncation and time-varying exposures for pregnant women who had their first ANC contact in the first trimester. Left truncation adjusts for the temporally changing risks of miscarriage as a pregnancy progresses and the varying gestation at presentation. All cases were censored at the gestation timepoint of miscarriage or at 28 weeks of gestation. Background and more details for this statistical approach are provided elsewhere [3,4].

The model to assess the association between monoinfections of hookworm, *A. lumbricoides* and *T. trichuria* were adjusted for the following factors: (i) STH categories, (ii) migration status, (iii) smoking, (iv) age, (v) gravidity, (vi) history of miscarriage, (vii) ethnicity, (viii) literacy status, (ix) anaemia at first HCT and (x) non-malarial fever 1^st^ trimester.

## Association of STH infections and stillbirth

To estimate the association between STHs and stillbirth, all pregnancies that ended in a miscarriage were excluded. A Cox proportional hazards model that accounted for left truncation with gestation as the time scale and non-informative right censoring at the time of pregnancy outcome (i.e. stillbirth or livebirth) was fitted to estimate the hazard ratio (HR).

The model to assess the association between monoinfections of hookworm, *A. lumbricoides*, *T. trichuria* were adjusted for the following factors: (i) STH categories, (ii) migration status, (iii) smoking, (iv) age, (v) gravidity, (vi) history of stillbirth, (vii) literacy status, (viii) anaemia in pregnancy.

## Association of STH infections and preterm birth

To estimate the association between STH infections and preterm birth (PTB), pregnant women at risk (defined as first antenatal care contact before completion of 37 weeks of pregnancy) and pregnancies ending in a livebirth were included in the analysis. Association of STH infections and PTB were estimated by fitting a logistic regression model that adjusted for the following factors: (i) STH categories, (ii) migration status, (iii) smoking, (iv) ethnicity, (v) age, (vi) gravidity (primigravida vs multigravida), (vii) previous PTB, (viii) underweight, (ix) GH/Chronic HTN and (x) preeclampsia/eclampsia.

## Association of STH infections and born too small for gestational age (SGA)

Cases with a live birth outcome were considered for the investigation of the association of STH infections and SGA. Small for gestational age (SGA) was defined as a birthweight below the 10^th^ centile, following gestation and sex adjusted birthweight centiles as published by the Intergrowth 21^st^ consortium [5]. Post term pregnancy outcomes (i.e. gestational age of more than 42^+6^ [weeks+days] at delivery), infants with an abnormal new-born exam, infants born too large for gestational age and neonates weighed after the first 72 hours after delivery were excluded from this analysis. Association of STH infections and SGA were estimated by fitting a logistic regression model that adjusted for the following factors: (i) STH categories, (ii) migration status, (iii) smoking, (iv) age, (v) gravidity (primigravida vs multigravida), (vi) literacy status, (vii) underweight, (viii) short stature, (ix) GH/Chronic HTN and (x) preeclampsia/eclampsia.

## Additional definitions for factors used for model adjustment

Pregnancy induced hypertension (PIH) was defined as systolic BP >140 mmHg or diastolic BP >90 mmHg, detected >20 weeks of gestation, in the absence of proteinuria; preeclampsia definition was based on the PIH definition and additionally required proteinuria; hypertension and proteinuria were measured at least two times and six hours apart. Eclampsia was based on the PIH definition with seizure activity.

|  |
| --- |

# References

1. Textor J, van der Zander B, Gilthorpe MS, Liskiewicz M, Ellison GT. Robust causal inference using directed acyclic graphs: the R package “dagitty.” Int J Epidemiol. 2016;45: 1887–1894. doi:10.1093/ije/dyw341

2. Moore KA, Fowkes FJI, Wiladphaingern J, Wai NS, Paw MK, Pimanpanarak M, et al. Mediation of the effect of malaria in pregnancy on stillbirth and neonatal death in an area of low transmission: observational data analysis. BMC Med. 2017;15: 98. doi:10.1186/s12916-017-0863-z

3. Meister R, Schaefer C. Statistical methods for estimating the probability of spontaneous abortion in observational studies - analyzing pregnancies exposed to coumarin derivatives. Reprod Toxicol Elmsford N. 2008;26: 31–35. doi:10.1016/j.reprotox.2008.06.006

4. Moore KA, Simpson JA, Paw MK, Pimanpanarak M, Wiladphaingern J, Rijken MJ, et al. Safety of artemisinins in first trimester of prospectively followed pregnancies: an observational study. Lancet Infect Dis. 2016;16: 576–583. doi:10.1016/S1473-3099(15)00547-2

5. Villar J, Cheikh Ismail L, Victora CG, Ohuma EO, Bertino E, Altman DG, et al. International standards for newborn weight, length, and head circumference by gestational age and sex: the Newborn Cross-Sectional Study of the INTERGROWTH-21st Project. Lancet. 2014;384: 857–68. doi:10.1016/s0140-6736(14)60932-6
