# Supplementary material for: Burden of soil-transmitted helminth infection in pregnant refugees and migrants on the Thailand-Myanmar border: Results from a retrospective cohort
Source: PLoS Negl Trop Dis. 2021 Mar 1;15(3):e0009219. doi: 10.1371/journal.pntd.0009219 (PMC7951971; doi:10.1371/journal.pntd.0009219)
Supplement: Attachment — Submitted filename: 4. PNTD-D-20-01995_Response.docx [file pntd.0009219.s007.docx]

**Brummaier T *et al.* “Burden of soil-transmitted helminth infection in pregnant refugees and migrants on the Thailand-Myanmar border: results from a retrospective cohort” (your reference no. PNTD-D-20-01995) – Point-by-point response**

First of all, we would like to take the opportunity to thank the reviewers for their invested time and constructive feedback to enhance the quality of this manuscript.

Please note that the line numbers provided here refer to the line numbers as they appear in the word document.

**Reviewer #1**:

*General comment
The article is well written and the experiment is well conducted but in my opinion this paper has an important drawback:*

**Response:** We would like to thank Reviewer#1 for the positive general comments and also for pointing out limitations of our study. The drawbacks are addressed in the point-by-point responses below. However, before going in *medias res*, we would like to clarify that the presented data and conclusion are not derived from an experiment designed to test a specific hypothesis, but rather real-world data that were routinely collected from antenatal care attendants over a 54-month period on the Thailand-Myanmar boarder.

*Reviewer#1 comment:*

*The authors do not recognize that the women population selected presents low level of STH infection (around 20%) principally due to A lumbricoides and therefore their experiment had no possibility to show any morbidity caused by the parasites.*

**Response:** We thank Reviewer #1 for this suggestion. We understand that this comment and the number of 20% is to be regarded in the context of the 2017 WHO guideline on preventive chemotherapy in at-risk population groups [1] that is mentioned below. First, we would like to point out that an observational study design is not appropriate to show causation; however, observational data can indicate associations. In fact, the data presented in this manuscript identified associations between soil-transmitted helminth infections and several outcomes (e.g., maternal anaemia or eosinophilia) for which causation is established in published literature. This is suggestive of the robustness of the data and that an association can be shown even if the prevalence is below an arbitrary cut-off of 20%. As a matter of fact, we are not sure, what the asserted population prevalence of around 20% necessary to show associations is based on. A population prevalence as high as 20% is rarely seen in any communicable and non-communicable diseases, yet morbidities of countless diseases with a significantly lower prevalence are known and confirmed. To provide an unrelated example: in an observational cohort study, loss of taste or smell was found to be significantly associated with SARS-CoV-2 seropositivity in a population with only 4% seroprevalence (= positivity) [2]. Therefore, to our understanding, an inference on morbidities associated with soil-transmitted helminth infections are more dependent on strength of the association (or causation in a different study design) rather than the disease prevalence.

*Reviewer#1 comment:*

*A population with so low STH prevalence of hookworm (1%-6%) and T. trichiura (2.4%) do not qualify for preventive chemotherapy according the WHO recommendations (please refers to WHO guidelines 2017:“ Preventive chemotherapy, using single-dose albendazole (400 mg) or mebendazole (500 mg), is recommended as a public health intervention for pregnant women, after the first trimester living in areas where both: (i) the baseline prevalence of hookworm and/or T. trichiura infection is 20% or higher among pregnant women, and (ii) anaemia is a severe public health problem, with a prevalence of 40% or higher among pregnant women.”)*

*The reason why PC is not recommended in populations of women where the prevalence of Hookworm and T. trichiura is lower than 20% is because at this level of prevalence the only infections present are the ones of light intensity that are not expected to cause morbidity.*

**Response:** This important information was added to the Discussion (**see revised manuscript, lines 363–366**) and the respective WHO reference was provided. Moreover, the conclusion was amended to make it clearer that this population does not meet the requirements for preventive chemotherapy, as per WHO recommendations. Instead, universal screening and treatment of positive pregnant women was recommended.

*Reviewer#1 comment:*

*I think that an experiment conducted in this population with so low pevalence had no possibility to demonstrate any morbidity caused by the parasites and therefore is probably unnecessary.*

**Response:** We kindly refer to the previous responses that address the fact that the presented data are derived from a routine antenatal care program rather than an experimental study and we tried to elaborate why we disagree with the notion that the prevalence of exposure reported is insufficient to demonstrate an association.

*Reviewer#1 comment:*

*However, there is a specific reason why suggest to reject this article despite the fact that I considered well written and resulting from a well conducted experiment:
Unfortunately, researchers that conduct systematic review are frequently not experienced on the specific disease but only on the methodology for conducting the review; as consequence, especially in the area of deworming, when the results of studies similar to the ones I am reviewing here (no possibility to demonstrate an effect because of the low prevalence) are summarized with the ones conducted in population where the level of parasites warrant intervention, they produce serious distortion of the results of the systematic review and therefore are leading to inapropriate conclusion.*

*This is why I consider this kind of articles not only unnecessary but counterproductive and therefore I reccomend rejection.*

**Response:** Here we also respectfully disagree with the conclusion reached by Reviewer #1, and our position is justified on multiple grounds.

First, the manuscript lays out in detail that this *real-world*, observational data derived from an active antenatal care program on the Thailand-Myanmar border where soil-transmitted helminths and other neglected tropical diseases prevail. It is clearly stated in the Method section that pregnant women were treated *in the event of a STH-positive sample* (**see revised manuscript, lines 184–187, unchanged from the original version**). This stands in clear contrast to *deworming* (i.e., preventive chemotherapy), which was not provided to the pregnant women reported here. In an effort to make this point clearer, the fact that no routine deworming was provided, was slightly expanded on the Introduction (**see revised manuscript, line 123**).

Second, when Reviewer #1 states “(…) when the results of studies similar to the ones (…) are summarized with the ones conducted in population where the level of parasites warrant intervention (…)”, one might conclude that pregnant women who are diagnosed with a soil-transmitted helminth infection but originate from a population with a low prevalence (or even a sporadic case from a non-endemic region) do not require treatment.

Third, the recommendation to reject the manuscript based on the possibility that in the future a third party may reach an inappropriate conclusion by misinterpreting the data, is beyond the authors, and likely the editors’ control.

Fourth, some of the authors have worked on the epidemiology and control of major helminthiases over the past 20 years, including original research on drug interventions, implementation and evaluation of cluster-randomized trials, etc., and hence, are well acquainted with the field of study and contemporary debate about systematic reviews and meta-analysis (see, for example, Andrews JR, Bogoch II, Utzinger J, The benefits of mass deworming on health outcomes: new evidence synthesis, the debate persists. Lancet Glob Health. 2017, [3]).

Against this background, no action has been taken.

********************************************************************************

**Reviewer #2:**

*Given the primary objectives of the study, it was expected that the evidence would be used to inform health policy and practice in the two study populations of migrants and refugees in terms of timing and frequency of antenatal care and public health interventions like deworming and iron supplementation.*

**Response:** As stated in the Abstract and toward the end of the Introduction, the “aim of this study was to assess the burden of STH infection in a pregnant population living on the Thailand-Myanmar border, and to compare the prevalence of different STH species between a mobile migrant population and a population confined to the largest refugee camp along the Thailand-Myanmar border” (**see revised manuscript, lines 39-42, unchanged from the original version and lines 122-125**). Reviewer #2 correctly points out below that the WHO recommends different methods (i.e., Kato-Katz technique) to assess the prevalence and intensity of soil-transmitted helminth infection in order to implement preventive chemotherapy programs. The limitations of the method used to assess the prevalence of soil-transmitted helminth infection in these two populations are elaborated in the manuscript. Informing public health policies solely based on the reported data may not be sufficient to warrant a change in the current public health interventions. However, the findings of the presented analysis are relevant to show that the detected prevalence of soil-transmitted helminth infection, based on a suboptimal – but widely used – diagnostic approach is not negligible and needs further investigation to appropriately inform potentially required changes in public health policies. In response to Reviewer #2’s valid comment, the Conclusion was extended in the sense that improved examination techniques would enable better informed policy decisions (**see revised manuscript, lines 448-450**).

*Reviewer#2 comment:*

*Given also the significant difference in the occurrence of STH infection in the two study populations, an appreciation of what is currently being provided in terms of antenatal care in the respective settings would be warranted and, if necessary, appropriate population-specific remedial action proposed. An analysis of relevant outcomes, again in the two populations separately, would seem the most beneficial in terms of subsequent health interventions that should be considered by health authorities. Therefore all analyses should, as a minimum, be disaggregated by population.*

**Response:** The extent of routine care that was provided to both populations is laid out in detail in the Methods (**see revised manuscript, lines 165-188, unchanged from the original version**). As mentioned in the manuscript, ANC operations in Mae La refugee camp ceased at the end of 2016 and were taken over by other NGOs. However, close cooperation with these NGOs exists and findings of this analysis will be shared with them. As raised by Reviewer #1, the reported prevalence of soil-transmitted helminth infection does not warrant preventive chemotherapy for the migrant population (see above). Hence, currently a systematic screening and treatment of positive cases is still adapted in the migrant population.

In regard to the request that all analysis should be disaggregated by population, we would like to point out that the following data was already presented disaggregated for the migrant and refugee population in the original manuscript: Demographic characteristics (Table 1), Time trend of infected pregnant women (Table S2) and Pregnancy outcomes associated with soil-transmitted helminth infection (Table S4). However, we do acknowledge that occasionally the pooled data was presented in the Results and/or Discussion section of the main body of the manuscript when the authors found it appropriate to do so.

Table S3 (Associations between anaemia at first ANC contact and a STH infection) was disaggregated as per Reviewer #2's suggestion. Due to space limitations, the original Table S3 was split into Table S3a (Associations between anaemia at first ANC contact and a STH infection in the Migrant population) and Table S3b (Associations between anaemia at first ANC contact and a STH infection in the Refugee population). **See revised Supporting Information Table S3a and Table S3b.**

*Reviewer#2 comment:*

*The rationale for undertaking exploratory analyses is missing. For example, an examination of the association between STH and anemia is not informative, as this association is well known and one would design a different study to examine such an association anyway (e.g. STH diagnosis would be based on a different test). The rationale for all exploratory analyses therefore should be clarified or the analyses should be removed.*

**Response:** As stated in the last sentence of the Introduction, an exploratory analysis was done to investigate associations between soil-transmitted helminth infection, maternal health, and pregnancy outcomes. We argue that this lays out the rationale for our analysis. Exploratory data analysis is a tool to detect potential associations which may lead to formulation of additional hypotheses that could then lead to new data collection and other, controlled experiments. Hence, the reported associations between – in particular soil-transmitted helminth infections and various adverse pregnancy outcomes – need confirmation. Limitations of such an exploratory analysis are laid out in the manuscript in the last sentence of the Conclusion (**see revised manuscript, lines 453-455, unchanged from the original version**). Reviewer #2 correctly indicates that the association between soil-transmitted helminth infection and anaemia is well known. However, there are several reasons to report this detail even though it is already known. First, the corroboration of this association is an indicator of the robustness of the data. This is of particular relevance as the reported stool examination technique is not optimal. Moreover, the magnitude of the effect of soil-transmitted helminth infection on anaemia prevalence is explored in this dataset and other relevant risk factors for anaemia at first ANC presentation are reported (disaggregated by the refugee and migrant population in the revised manuscript). Also, when considering other comments made by Reviewer #2 above (“(…) the evidence would be used to inform health policy and practice (…) timing and frequency of antenatal care and public health interventions like deworming and iron supplementation”), it seems important to understand the prevalence of anaemia and the association with soil-transmitted helminth infection or other factors (e.g., late presentation to ANC). Estimated population attributable fractions (PAFs), as reported in Tables S3a and Table S3b in the revised manuscript, provide a measure what effect can be expected from interventions such as early ANC enrolment (concomitant with supplements), deworming of positive cases, etc. We would therefore argue to keep the association between maternal anaemia and soil-transmitted helminth infections in the manuscript.

*Reviewer#2 comment:*

*Retrospective data can certainly be used to explore the study’s primary objectives. But there are limitations to this type of data. Selection bias is one such limitation. Selection bias here can occur from missing important numbers of pregnant women in the database, or limiting the numbers of women in certain analyses because of incomplete data. To give one example, as ANC was known to only be fully operational at the end of 2013 in the study populations until the end of 2016, why were data to be analyzed not restricted to the three full years of 2014, 2015 and 2016? It should be kept in mind that the ‘prevalence’ reported in this study is a 54-month cumulative prevalence. It would be helpful to annualize this figure. It might then be clarified whether STH is, or is not, of public health importance in these populations and that relevant health policy and practice should be reviewed. Also, as migrant and refugee populations were found to be significantly different in terms of STH infection (despite the measurement error), all analyses should be performed for each of these study populations separately.*

**Response:** We thank Reviewer #2 for pointing out the critical limitations of retrospective datasets. To maximize transparency, data from excluded women were added and compared to data from included women (compare Table S1). Moreover, the limitations of retrospective analysis of observational data were addressed in the manuscript (**see revised manuscript, line 427, unchanged from the original version**).

Annualised data on the prevalence of soil-transmitted helminth infections are presented in the Supporting information (Table S2). A footnote was added to re-emphasise that a full set of data was not available for the year 2013 and 2017. Moreover, pooled numbers for the annual prevalence for the years with a complete set of data (i.e., 2014-2016) was added to the revised manuscript and Table S2 was referenced to indicate to the reader where additional information is available (**see revised manuscript, lines 285–289**).

*Reviewer#2 comment:*

*The question of trimester is always problematic. WHO defines the first 16 weeks of pregnancy as the first trimester, so why was 14 weeks used in this study (line 200)? (WHO. Integrated Management of Pregnancy and Childbirth. Pregnancy, Childbirth, Postpartum and Newborn Care: A guide for Essential Practice. WHO, 2015; see page 180.) At a minimum, the data need to re- analyzed with the WHO definition of trimester. The definition of trimester is important as this will affect the estimate of the number of miscarriages (line 200). All instances referring to trimester in the text and tables require revision.*

**Response:** We agree with Reviewer #2 that the question of trimester definition is critical. It is correct that in the referenced WHO document (WHO, 2015. Integrated management of pregnancy and childbirth. Pregnancy, childbirth, postpartum and newborn care. A guide for essential practice (3rd edition), [4]), there is a statement on page 180 that for this particular guide the duration of the first trimester was expressed by less than 4 months or less than 16 weeks. A rationale for choosing this particular cut-off is not provided. However, according to our understanding it cannot be concluded that this is the official WHO definition of the first trimester of pregnancy, and we would like to take the opportunity to elaborate why.

First, the WHO published recommendations on ANC for a positive pregnancy experience in 2018 (published after the guide cited by Reviewer#2) with a clear recommendation to start intermittent preventive treatment for malaria in pregnancy at ≥13 weeks of pregnancy (page 2) and specifies in the same document that “Pregnant women in malaria-endemic areas should start IPTp-SP as early as possible in the second trimester (i.e., 13 weeks)” (page 7) [5].

Second and in reference to the presented topic of soil-transmitted helminthiasis in pregnancy, in 2017 (published after the guide cited by Reviewer #2), the WHO published guidelines for preventive chemotherapy to control soil-transmitted helminth infections in at-risk population groups [1]. These recommendations for deworming pregnant women are based on the results from a systematic review from the Cochrane Pregnancy and Childbirth Group [6]. This systematic review compiles evidence from studies where the first trimester of pregnancy was defined as <14 weeks (Torlesse 2001 [7]), inclusion of second trimester (≥18 weeks; < 26 weeks) pregnant women (Larocque 2006 [8]), inclusion of second trimester (≥16 weeks of gestation at first ANC booking) pregnant women (Ndyomugyenyi 2008 [9]) and no mention of weeks of gestation at all (Elliot 2005 [10]). Hence, it does not appear to the authors that the WHO has an unequivocal definition of the first trimester of pregnancy.

Lastly, no definition of pregnancy trimesters was found on the official WHO homepage.

Other reasons for not changing the definition of the first trimester:

- Consistency with other papers recently published in PLoS Neglected Tropical Diseases (e.g., Souza *et al*, 2021, PLoS Negl Trop Dis [11] or Hay *et al*, 2018, PLoS Negl Trop Dis, [12].
- The following professional associations define the first trimester of pregnancy divergent from the definition proposed by Reviewer #2 in the sense that earlier than 16 weeks cut-offs are published on their webpages:
  - The American College of Obstetricians and Gynecologists;
  - Royal College of Obstetricians & Gynaecoloists; and
  - The Royal Australian and New Zealand College of Obstetricians and Gynaecologists.
- The reported data are derived from an antenatal care program that does define the first trimester of pregnancy as <14 weeks of gestation; this entails that the routine management of pregnant women is based on this definition. For instance, extending the first trimester to 16 weeks would add a potential bias derived from anthelmintic treatment, which was provided to pregnant women with a positive stool test in the EGA bracket from ≤14 to <16 weeks of gestation. Moreover, while extension of the first trimester to 16 weeks of gestation may slightly increase the number of miscarriages, the authors do not see why pregnant women enrolled in the ANC program between from ≤14 to <16 weeks of gestation would have a different ratio of soil-transmitted helminth positive and negative cases.
- In embryology, the first trimester ends with Carnegie Stage 23 at a gestational age of 10 weeks, which does also not support the 16 weeks cut-off proposed by Reviewer#2 [13].
- The verbatim definition of *trimester* (i.e., *a period of three months*) does not support a 16-week cut-off for the first trimester.

Take therefore, we respectfully disagree to re-analyse all data based on the first trimester cut-off proposed by Reviewer#2.

*Reviewer#2 comment:*

*The measurement of the STH infection itself is problematic and subject to measurement error. WHO recommends that the Kato- Katz method be used to assess the prevalence and intensity of STH infection in order to then implement a preventive chemotherapy program in endemic areas. As a non-standardized diagnostic test was used in this study, with unknown estimates of species-specific sensitivity and specificity, it is difficult to interpret the results. Therefore, the accuracy of the estimates of prevalence and intensity for monoinfections, or the prevalence of multiple species infections, is unknown, and any analysis using these figures is subject to measurement error. This is one of the reasons for dropping the exploratory analyses. It is unclear why the 6.3% due to multiple stool testing from the same individuals was not removed from all analyses...They bias the results if they are not removed.*

**Response:** Reviewer #2 is correct that the examination method used to detect soil-transmitted helminth infections is not the preferred and recommended method by WHO and that other, perhaps more sensitive tests exist. This fact is elaborated in the manuscript and pointed out as a limitation. However, while the used examination method is not ideal, concentration by flotation techniques, which allow separation of parasitic elements from organic debris are commonly used and are part of the WHO bench aids for diagnosis of parasitic infections [14]. Moreover, the reported test was indeed used in a standardised manner, as it was administered and scored in a consistent way by following standard operating procedures (SOPs). We do acknowledge that the accuracy suffers from a suboptimal test and lay this out in the manuscript. In addition, the Conclusion was amended to indicate that a more sensitive testing system is desired to inform public health measures (**see revised manuscript, lines 448-450**). As mentioned above, the exploratory analysis, was done to detect potential associations that may lead to formulation of additional hypotheses and lead to controlled experiments with adequate examination methods. Context for interpretation of these exploratory analyses was provided in the manuscript.

In reference to the comment that multiple testing biases the results, we argue that multiple testing has been found to be a suitable approach to enhance the diagnostic accuracy and the examination of multiple stool samples has been recommended for accurate diagnosis of intestinal parasitic diseases [15]. In particular when considering presented data on pregnancy outcomes and the fact that all tests were done before the outcome, any positive case is relevant for outcome assessment. However, based on the Reviewer #2's comment, the data of cases with multiple testing was explored and the following was found: the vast majority of participants with multiple tests were tested negative multiple times. Hence, while no effect from participants with repeated negative samples on the result can be expected, the likelihood of a true negative is reinforced by multiple testing. Moreover, the reported models investigating adverse pregnancy outcomes were run with cases removed who had multiple stool tests and the direction of associations remained the identical for all outcomes. This additional finding was indicated in the Discussion of the revised manuscript (**see revised manuscript, lines 424-426**). In summary, we respectfully disagree with the notion that multiple testing introduces a bias, in fact it enhances the diagnostic accuracy. Cases with multiple test were therefore not removed from this revised version of the manuscript.

*Reviewer #2:*

*All results tables, figures and supplementary material need to be reconsidered given the above comments.*

**Response:** Thank you for the suggestion. Please note that tables, figures, and supporting information were amended, where authors were in agreement with the reviewer’s assessment.

*Reviewer #2:*

*The discussion also then should be focussed on the primary objectives to inform public health interventions to the two study populations. There is considerable repetition of results in the discussion which can be removed. All mention of benzimidazole efficacy should be removed as this was not adequately investigated in this study.*

**Response:**

Reviewer #2 suggests changing the narrative of the Discussion (e.g., focus on the primary objectives to inform public health interventions”) and removing “considerable repetitions of results in the discussion” section. As stated in the in last paragraph of the Introduction, the “aim (…) was to assess the burden of STH infection (…), and to compare the prevalence (…) between a mobile migrant population and a population confined to the (…) refugee camp (…)”. We now tried our level best to lay out that the reported data are only suitable to a limited extend to inform public health policies. However, it is a foundation that illustrates that the STH burden in both populations (i.e., migrants and refugees) are not negligible and further investigation, with more sensitive diagnostic technique, is warranted. In the Discussion, we attempted to put the findings into context of what is known from published literature, peculiarities from both populations, and recommendation for further steps to be undertaken. We do hope that this satisfies Reviewer #2 and meets the journal expectations. Regarding the “considerable repetitions of results in the discussion” section, we would like to emphasize again that we tried our level best to put results into a broader context in the Discussion. In the current form, we do not see "considerable repetitions of results"; however, we would appreciate if the Reviewer could indicate inappropriate repetitions and we would happily to remove them.

It is correct that benzimidazole efficacy was not formally investigated. Among others, it is offered as a potential explanation for the discrepancy between the *A. lumbricoides* burden in the two populations as benzimidazoles are routinely used for deworming children (not adults or pregnant women) in the refugee camp, while no such exposure is present in the migrant population. It is also fully disclosed that “(…) no formal investigation on selective pressures and the efficacy of benzimidazole for treatment of STH infection in the area has been done.” Hence, we would like to keep the respective text as is.

*Reviewer #2:*

*This manuscript details a retrospective review of cumulative data (recorded between 2013 and 2017) from screening for STH infection among migrant and refugee pregnant women living in camps along the Thai-Myanmar border. It is actually unclear why the study’s objective was to describe and compare STH infection between these two study populations. Describe yes, but why compare? Would results influence screening procedures or deworming policy/practice? If this was the intent, it needs to be made clearer.*

**Response:** We thank Reviewer #2 for this suggestion. While both populations originate from a similar background (e.g., distribution of ethnicities) and comparable demographics (e.g., age, gravida, etc.), the STH prevalence was significantly different between refugee (30.8%) and migrant (16.7%) populations. The results indicate that both populations have different exposure and may require different interventions. However, we agree that comparing both groups does not add value to the interpretation of the distinct results and consequences to be drawn. We therefore reduced the aim to describing the burden in both populations. Nonetheless, disaggregation of all analyses by population, as suggested by Reviewer#2, will intuitively tempt the reader to compare results between both populations.

********************************************************************************

**Reviewer #3**:

*The study population, objectives, design and ethics are well described and appropriate statistical analyses are employed to support their conclusions.*

**Response:** We thank Reviewer#3 for the positive and favourable general assessment.

*Reviewer #3: Results are clearly presented in line with the study objectives. However, I identified a few issues below, these need to be addressed*

1. *A couple of numbers in the text do not match what is indicated in the tables, let the authors be consistent with this.*
2. *Lines 256 – 258: STH infections were detected earlier... The numbers on line 257 e.g. 14+2(9+0 – 22+4) do not match the numbers in Table 1*

**Response:** The numbers presented in the text refer to the EGA of enrolment to the ANC programme, while the numbers in Table 1 reflect the EGA when the stool sample was provided. This was clarified in the revised manuscript (**see revised manuscript, lines 276-279**).

3. *Table S2: what is the motivation for showing STH neg while for the other mono infections it shows positivity? Wouldn’t it be better if all infections showed the same thing (either positivity or negativity)?*

**Response:** Women had a stool examination that was either negative or was positive with a mono-infection, so it does show what the reviewer suggests.

4. *Lines 269 – 281: The denominators are 4041 and 8701 for refugee and migrant populations respectively. This contradicts the figures in Table 1 i.e. 3909 and 8517 respectively. Please ensure that this is well explained.*

**Response:** We thank Reviewer #3 for noticing this. The discrepancy derives from the fact that in Table 1, multiple infections (i.e., 184 in the migrant population and 132 in the refugee population) are not shown. A note was added to Table 1 that the multiple infections are not shown in the Table (**see revised manuscript, Table 1**).

5. *Line 289: In an adjusted logistic regression model... I think that the potential confounders adjusted for need to be mentioned, perhaps in the methods section.*

**Response:** The confounders that were adjusted for in the model investigating the association between soil-transmitted helminth infection and anaemia at the first ANC presentation were added to the Methods section (**see revised manuscript, lines 231-234**).

6. *Lines 293 – 294: would you consider a trend test to confirm the result that the intensity of infection was associated with anaemia?*

**Response:** The association between the intensity of infection and anaemia is reported in detail in Table S3 (Table S3a and Table S3b in the revised manuscript). The effect of rare, low, medium and high infection intensity on the odds that pregnant women present with anaemia were explored and are reported at the bottom of the table(s). We do hope that this addresses the Reviewer's comment appropriately. In any case, if the Reviewer envisioned different testing to explore the trend between anaemia and intensity of infection, we would appreciate if more details could be provided.

7. *Lines 307 – 309: in an unadjusted analysis... you have reported adjusted analyses before, why not for this result also?*

**Response:** There is already a considerable amount of information in the manuscript. The association with malaria is not the focus of the paper, and hence, we prefer not to do a complete adjusted analysis.

8. *Please make Figure 3 clearer, it looks fuzzy at the moment*

**Response:** Figures were uploaded to the PLoS Neglected Tropical Diseases editorial manger system following the journal requirements (i.e., resolution of 300 pixels per inch). However, from experience we know that occasionally compiled documents shared with reviewers often compress figures which leads to suboptimal quality. Can the production editors provide an option to access and download the original file?

9. *Lines 315 – 316: when adjusting for confounders, ... please mention these confounders in the methods section. I know they appear in supplementary information but it might be better to include them in the main text.*

**Response:** Adjusted confounders for all models investigating the association between soil-transmitted helminth infection and birth outcome were added the Methods section of the main text (**see revised manuscript, lines 238-252**).

*Reviewer #3: Conclusions are supported by the data and limitations are largely well described, save for the statistical power for association analyses.
Public health relevance is addressed, though I think a bit more of this needs to appear in the abstract!*

**Response:** As suggested, the Abstract was extended and relevance to public health was added (**see revised manuscript, lines 37-38**).

*Reviewer #3: This is a very well-written paper about the burden of STH infection in a pregnant population living on the Thailand- Myanmar border. Authors compare the prevalence of different STH species between a migrant population and a population of refugees in a camp, they also report an exploratory analysis on associations between STH infection, maternal health and pregnancy outcomes.*

*I think though, that the authors need to acknowledge the limitation of lack of statistical power for the exploratory association analyses or else confirm that this is not a problem.*

**Response:** We thank Reviewer #3 for this comment. The exploratory analysis does have its limitations and we tried to emphasise these more clearly (**see revised manuscript, lines 453-456**). Further investigation by using different method of hypothesis testing is warranted.

**References:**

1.	World Health Organization (2017). Guideline: preventive chemotherapy to control soil-transmitted helminth infections in at-risk population groups. Available from: https://www.who.int/nutrition/publications/guidelines/deworming/en/ (accessed 4 January 2021).

2.	Iversen K, Bundgaard H, Hasselbalch RB, Kristensen JH, Nielsen PB, Pries-Heje M, et al. Risk of COVID-19 in health-care workers in Denmark: an observational cohort study. The Lancet Infectious Diseases. 2020;20: 1401–1408. doi:10.1016/S1473-3099(20)30589-2.

3.	Andrews JR, Bogoch II, Utzinger J. The benefits of mass deworming on health outcomes: new evidence synthesis, the debate persists. The Lancet Global Health. 2017;5: e4–e5. doi:10.1016/S2214-109X(16)30333-3.

4.	World Health Organization (2015). Integrated management of pregnancy and childbirth. Pregnancy, childbirth, postpartum and newborn care. A guide for essential practice (3rd edition). Available at: https://www.who.int/maternal_child_adolescent/documents/imca-essential-practice-guide/en/ (accessed 5 January 2021).

5.	World Health Organization (2018). WHO recommendations on antenatal care for a positive pregnancy experience. Highlights and key messages. Available at: https://www.who.int/reproductivehealth/publications/anc-positive-pregnancy-experience-summary/en/ (accessed 5 January 2021).

6.	Salam RA, Haider BA, Humayun Q, Bhutta ZA. Effect of administration of antihelminthics for soil‐transmitted helminths during pregnancy. Cochrane Database of Systematic Reviews. 2015 [cited 14 Oct 2019]. doi:10.1002/14651858.CD005547.pub3.

7.	Torlesse H, Hodges M. Albendazole therapy and reduced decline in haemoglobin concentration during pregnancy (Sierra Leone). Trans R Soc Trop Med Hyg. 2001;95: 195–201. doi:10.1016/s0035-9203(01)90164-6.

8.	Larocque R, Casapia M, Gotuzzo E, MacLean JD, Soto JC, Rahme E, et al. A double-blind randomized controlled trial of antenatal mebendazole to reduce low birthweight in a hookworm-endemic area of Peru. Trop Med Int Health. 2006;11: 1485–1495. doi:10.1111/j.1365-3156.2006.01706.x.

9.	Ndyomugyenyi R, Kabatereine N, Olsen A, Magnussen P. Efficacy of Ivermectin and Albendazole Alone and in Combination for Treatment of Soil-Transmitted Helminths in Pregnancy and Adverse Events: A Randomized Open Label Controlled Intervention Trial in Masindi District, Western Uganda. The American Journal of Tropical Medicine and Hygiene. 2008;79: 856–863. doi:10.4269/ajtmh.2008.79.856.

10.	the “Mother and Baby” study team, Elliott AM, Namujju PB, Mawa PA, Quigley MA, Nampijja M, et al. A randomised controlled trial of the effects of albendazole in pregnancy on maternal responses to mycobacterial antigens and infant responses to bacille Calmette-Guérin (BCG) immunisation [ISRCTN32849447]. BMC Infectious Diseases. 2005;5. doi:10.1186/1471-2334-5-115.

11.	Souza JP, Méio MDBB, de Andrade LM, Figueiredo MR, Gomes Junior SC, Pereira Junior JP, et al. Adverse fetal and neonatal outcomes in pregnancies with confirmed Zika Virus infection in Rio de Janeiro, Brazil: A cohort study. Lacerda MVG, editor. PLOS Neglected Tropical Diseases. 2021;15: e0008893. doi:10.1371/journal.pntd.0008893.

12.	Hay JA, Nouvellet P, Donnelly CA, Riley S. Potential inconsistencies in Zika surveillance data and our understanding of risk during pregnancy. Marques ETA, editor. PLOS Neglected Tropical Diseases. 2018;12: e0006991. doi:10.1371/journal.pntd.0006991

13.	Hill, M.A. Embryology. Timeline human development. Available at: https://embryology.med.unsw.edu.au/embryology/index.php/Timeline_human_development#First_Trimester (accssed 9 January 2021).

14.	World Health Organization (2019). Bench Aids for the diagnosis of intestinal parasites. Second edition. Available at: https://www.who.int/intestinal_worms/resources/9789241515344/en/ (accessed 11 January 20219).

15.	Knopp S, Mgeni AF, Khamis IS, Steinmann P, Stothard JR, Rollinson D, et al. Diagnosis of Soil-Transmitted Helminths in the Era of Preventive Chemotherapy: Effect of Multiple Stool Sampling and Use of Different Diagnostic Techniques. Engels D, editor. PLoS Negl Trop Dis. 2008;2: e331. doi:10.1371/journal.pntd.0000331.
